# Syngeneic mouse model of YES-driven metastatic and proliferative hepatocellular carcinoma

**DOI:** 10.1242/dmm.050553

**Published:** 2024-07-25

**Authors:** Laure Voisin, Marjorie Lapouge, Marc K. Saba-El-Leil, Melania Gombos, Joaquim Javary, Vincent Q. Trinh, Sylvain Meloche

**Affiliations:** ^1^ Institute for Research in Immunology and Cancer, Montreal, Quebec H3T 1J4, Canada; ^2^ Centre de recherche du Centre hospitalier de l'Université de Montréal (CRCHUM), Montreal, Quebec H2X 0A9, Canada; ^3^ Department of Pathology and Cell Biology, Université de Montréal, Montreal, Quebec H3C 3J7, Canada; ^4^ Molecular Biology Program, Faculty of Medicine, Université de Montréal, Montreal, Quebec H3C 3J7, Canada; ^5^ Department of Pharmacology and Physiology, Université de Montréal, Montreal, Quebec H3C 3J7, Canada

**Keywords:** Hepatocellular carcinoma, Tyrosine kinase, SRC family kinases, Cancer mouse model

## Abstract

Hepatocellular carcinoma (HCC) is a disease of high unmet medical need that has become a global health problem. The development of targeted therapies for HCC has been hindered by the incomplete understanding of HCC pathogenesis and the limited number of relevant preclinical animal models. We recently unveiled a previously uncharacterized YES kinase (encoded by *YES1*)-dependent oncogenic signaling pathway in HCC. To model this subset of HCC, we established a series of syngeneic cell lines from liver tumors of transgenic mice expressing activated human YES. The resulting cell lines (referred to as HepYF) were enriched for expression of stem cell and progenitor markers, proliferated rapidly, and were characterized by high SRC family kinase (SFK) activity and activated mitogenic signaling pathways. Transcriptomic analysis indicated that HepYF cells are representative of the most aggressive proliferation class G3 subgroup of HCC. HepYF cells formed rapidly growing metastatic tumors upon orthotopic implantation into syngeneic hosts. Treatment with sorafenib or the SFK inhibitor dasatinib markedly inhibited the growth of HepYF tumors. The new HepYF HCC cell lines provide relevant preclinical models to study the pathogenesis of HCC and test novel small-molecule inhibitor and immunotherapy approaches.

## Teaser

**Summary:** A mouse model of YES kinase-driven hepatocellular carcinoma recapitulates the pathophysiological features of clinically aggressive metastatic forms of this cancer and is useful for the preclinical evaluation of hepatocellular carcinoma therapies.

## INTRODUCTION

Liver cancer is the sixth most common cancer worldwide and the third leading cause of cancer-related deaths, with hepatocellular carcinoma (HCC) accounting for 80-90% of primary tumors (Llovet et al., 2021; Rumgay et al., 2022; Villanueva, 2019). It is one of the most lethal malignancies with a 5-year survival rate of ∼18%, second worst to pancreatic cancer. Most patients with HCC are diagnosed at advanced stages, when few therapeutic options are available (Llovet et al., 2021; Yang et al., 2019). The multi-kinase inhibitors sorafenib (Cheng et al., 2009; Llovet et al., 2008) and lenvatinib (Kudo et al., 2018) and the combination treatment atezolizumab/bevacizumab (Finn et al., 2020) are the only approved first-line treatments for patients with advanced-stage HCC, with limited benefit in overall survival. There is, therefore, a need for the development of new therapies and the identification of predictive biomarkers in HCC. At the molecular level, HCC can be classified into two major subclasses: a proliferation subgroup, characterized by activation of mitogenic signaling pathways, chromosomal instability, progenitor cell features and an aggressive clinical behavior; and a non-proliferation subgroup, associated with frequent WNT signaling activation, higher degree of differentiation and better outcome (Llovet et al., 2015; Zucman-Rossi et al., 2015).

Establishment of relevant preclinical mouse cancer models is crucial to the development and optimization of new therapeutics (Day et al., 2015). A variety of strategies have been used to model HCC *in vivo*, including development of genetically engineered mouse models (GEMMs), exposure to chemical hepatocarcinogens, special diet administration, heterotopic or orthotopic transplantation of human and mouse HCC cell lines, and patient-derived tumor xenografts (Bakiri and Wagner, 2013; Brown et al., 2018; Chen and Calvisi, 2014; Gu and Lee, 2022; He et al., 2015; Li et al., 2011). Each of these models has strengths and limitations and there is no perfect model. GEMMs can recapitulate the anatomical location and histopathological features of specific oncogene-driven HCCs, but are highly resource intensive, often show long latency periods and are not suitable for screening purposes. Chemical carcinogen- and diet-induced hepatocarcinogenesis is experimentally simpler than using GEMMs, but it is difficult to standardize and also shows long latency. As for other cancer types, subcutaneous implantation of human HCC cell lines in immunodeficient mice is the most common approach for preclinical therapeutic studies. These cancer models are relatively easy to use, are reproducible and are suitable for proof-of-principle drug testing studies. A recent comprehensive molecular profiling of a panel of human liver cancer-derived cell lines has highlighted the translational potential of these models (Caruso et al., 2019). However, many human HCC cell lines fail to grow in immunodeficient mice and, most critically, xenograft models preclude evaluation of anti-tumor immune responses. It is therefore important to continue to develop advanced HCC mouse models in order to select the most appropriate models for specific preclinical studies.

We have recently reported the identification of the SRC family kinase (SFK) YES (encoded by *YES1*) as a driver of HCC development in mice (Guégan et al., 2022). SFKs are a family of non-receptor tyrosine kinases composed of eight members in humans (Parsons and Parsons, 2004). They play important roles in transducing signals from multiple receptors to regulate cell proliferation, differentiation, survival, motility, angiogenesis and immune responses (Kim et al., 2009; Parsons and Parsons, 2004; Thomas and Brugge, 1997). Deregulated expression or activity of YES is frequently observed in human cancers and is associated with tumor development and metastatic progression (Lapouge and Meloche, 2023). We showed that YES is both necessary and sufficient to induce liver tumorigenesis in mice and identified the transcriptional co-activators YAP/TAZ as key effectors of YES transformation. The YES–YAP/TAZ signaling pathway is dysregulated in a subset of patients with HCC, predicting increased tumor burden and poor outcome (Driskill and Pan, 2021; Garmendia et al., 2022; Lapouge and Meloche, 2023; Shi et al., 2023; Zhang and Zhou, 2019).

In this study, we report the establishment of YES-driven mouse syngeneic HCC cell lines that recapitulate the pathophysiological features of aggressive proliferation class HCC. The newly derived HCC cell lines are capable of forming ectopic or orthotopic liver tumors that lead to intrahepatic and extrahepatic metastases to lung and lymph nodes. These models should prove useful to study the molecular pathogenesis of metastatic HCC and to evaluate the efficacy of novel therapeutic modalities.

## RESULTS

### Establishment of syngeneic HepYF cell lines from liver tumors of *YES1* transgenic mice

**Fig. 1.**
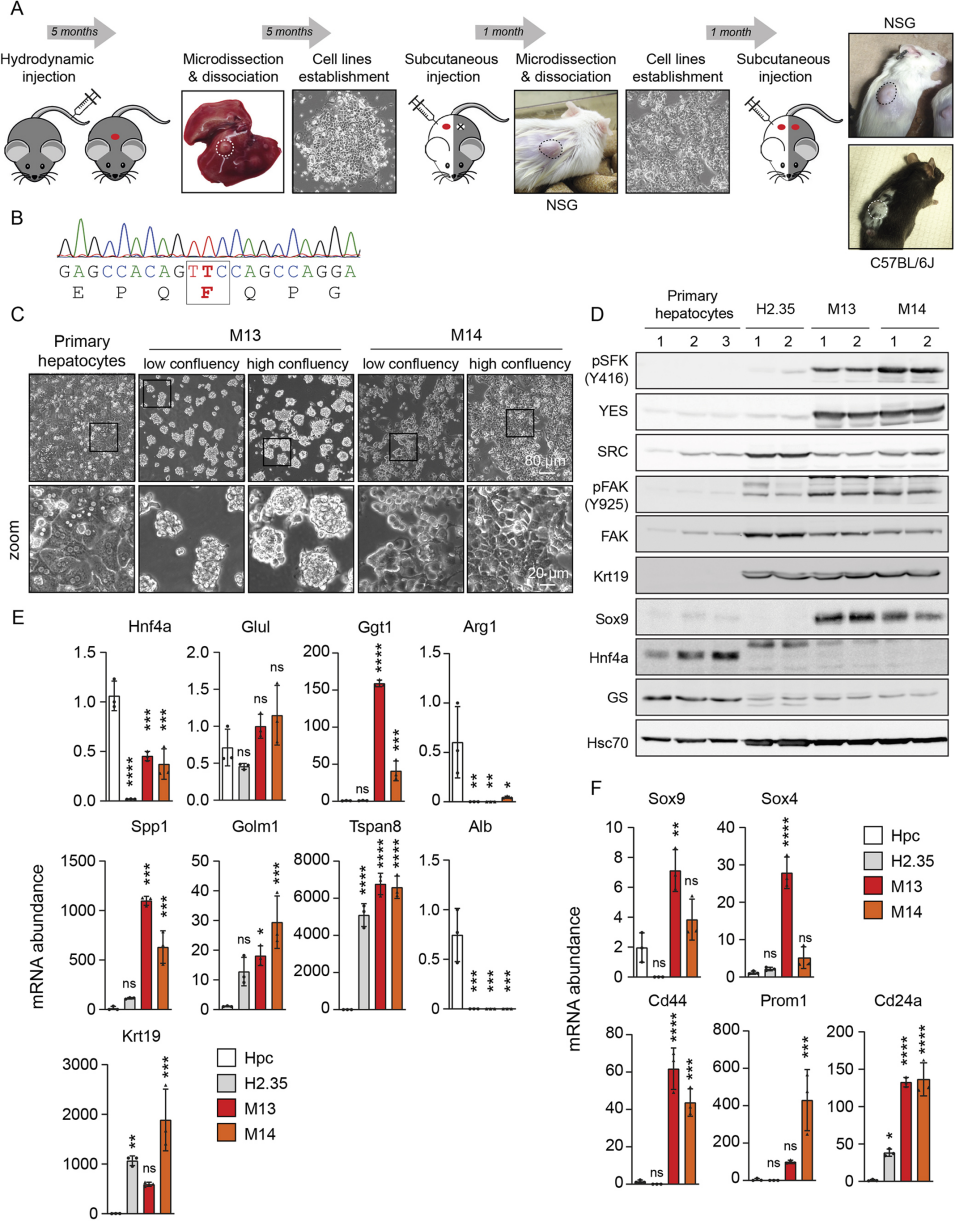
**Generation and phenotypic characterization of HepYF liver cancer cell lines.** (A) Schematic design of the establishment of syngeneic liver cancer cell lines from *YES1* Y537F transgenic mice. (B) Chromatogram of the genomic DNA sequence from HepYF-M13 cells confirming the Y537F mutation in the *YES1* gene. (C) Photomicrographs of HepYF-M13 and HepYF-M14 cell lines and comparison with mouse primary hepatocytes. Images are representative of more than three independent experiments. (D) Western blot analysis of SFK signaling and liver cancer markers in primary mouse hepatocytes, the immortalized H2.35 mouse hepatocyte cell line and HepYF cell lines. GS, glutamine synthetase (encoded by *Glul*). The numbers above the lanes represent individual biological replicates. (E,F) Quantitative PCR (qPCR) analysis of hepatocyte and liver cancer markers (E) and stem cell markers (F) in primary mouse hepatocytes (Hpc), H2.35 cells and HepYF cell lines. Data are mean±s.d. (*n*=3). *P*-values were calculated by one-way ANOVA with Tukey's post hoc test. ns, not significant; *P<0.05; **P<0.01; ***P<0.005; and ****P<0.001, compared to primary hepatocytes.

To develop novel syngeneic tumor models representative of SFK-driven HCC, we hydrodynamically injected C57BL/6J mice with plasmids encoding constitutively active human YES Y537F and Sleeping Beauty transposase to stably express the kinase in adult hepatocytes. We have previously reported that transgenic expression of activated YES Y537F in mouse hepatocytes induces liver tumor formation with high penetrance (Guégan et al., 2022). The mice were sacrificed after 5 months, the liver was removed, and the primary tumors were micro-dissected and processed for *ex vivo* culturing as described in the Materials and Methods. Tumor cell explants initially expanded as heterogeneous cell mixtures, but more homogenous epithelial-like cells eventually appeared after subculturing for multiple passages. Starting from three to four liver tumor fragments isolated from two *YES1* Y537F transgenic mice, we successfully derived six cell lines (Fig. 1A; Table S1). To evaluate their tumorigenic properties, the cell lines were injected subcutaneously into the flank of NSG and C57BL/6J mice. Growing tumors were observed for five of six cell lines in NSG mice but none of the cell lines engrafted in C57BL/6J mice. Allograft tumors from NSG mice were then excised and tumor cells were dissociated and expanded for five to six passages *in vitro*, prior to re-injection in NSG and C57BL/6J mice. All cell lines (referred to as HepYF cell lines) formed tumors in NSG mice, and the five lines developed tumors in syngeneic C57BL/6J hosts (Fig. 1A; Table S1). We confirmed by DNA sequencing that the cell lines express the *YES1* Y537F mutation (Fig. 1B). The newly derived HepYF-M13 and HepYF-M14 cell lines were further characterized in this study.

### Molecular characterization of HepYF cell lines

HepYF-M13 and HepYF-M14 cell lines showed a polygonal, epithelial cell morphology (Fig. 1C). Specifically, HepYF-M13 cells mainly grew as cellular aggregates in culture, whereas HepYF-M14 cells displayed mesenchymal-like features. As anticipated, the two liver tumor cell lines expressed higher levels of YES and showed a marked increase in SFK-activating phosphorylation compared to primary mouse hepatocytes and the immortalized H2.35 mouse hepatocyte cell line (Fig. 1D). Consistent with the activation of YES, we also observed an augmented phosphorylation of focal adhesion kinase (FAK, also known as PTK2) on Y925, a known SFK phosphorylation site (Schlaepfer and Hunter, 1996).

We measured the expression of a selected set of known hepatocyte and liver cancer markers by quantitative PCR (qPCR) analysis. HepYF-M13 and HepYF-M14 cells expressed HCC markers such as *Hnf4a*, *Glul*, *Ggt1*, *Spp1*, *Golm1* and *Tspan8*, and did not express the well-differentiated HCC markers *Arg1* and *Alb* (Fig. 1E). Expression of *Gpc3* and *Cps1*was below the limit of quantification. The two cell lines also expressed *Krt19*, a marker typically associated with cholangiocarcinoma, which is a recognized finding in highly aggressive HCCs with poor prognosis (Govaere et al., 2014). Reminiscent of human liver cancers that express both lineages (Brunt et al., 2018), we noted the upregulated expression of stemness markers such as *Sox9*, *Sox4*, *Cd44*, *Prom1* and *Cd24a* in HepYF-M13 and HepYF-M14 cells compared to their expression in normal hepatocytes and H2.35 immortalized hepatocytes (Fig. 1F). The upregulated expression of Krt19 and Sox9 was confirmed by western blot analysis (Fig. 1D).

**Fig. 2.**
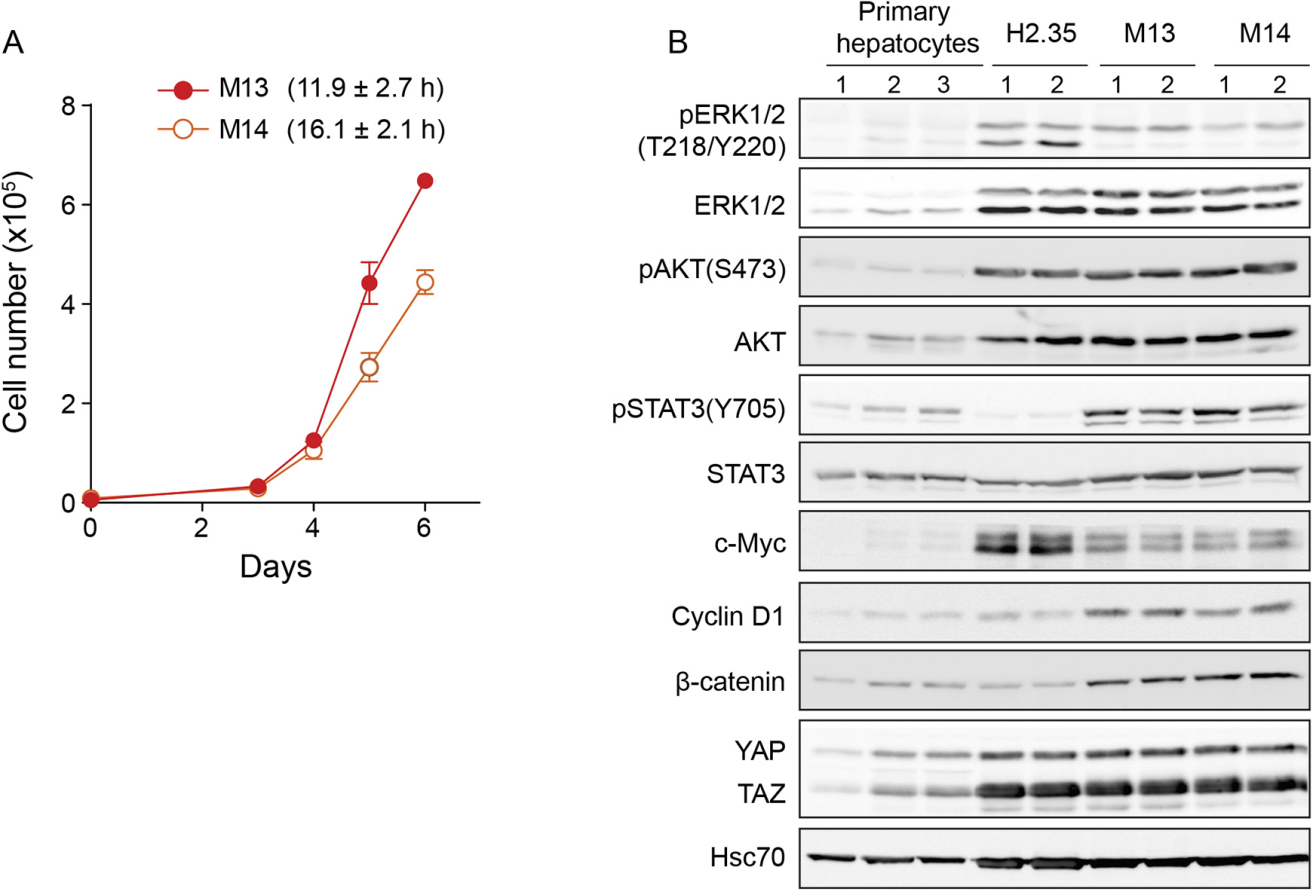
**HepYF cell lines are highly proliferative.** (A) Proliferation curves of HepYF-M13 and HepYF-M14 cell lines. Cellular doubling time was estimated during the exponential phase of cell division and corresponds to the time in hours required for the population of viable cells to double. Data show mean±s.e.m. and values in parentheses represent the mean±s.e.m. of doubling time (*n*=3). (B) Western blot analysis of oncogenic signaling pathways in primary hepatocytes, immortalized H2.35 cells and HepYF cell lines. The same set of samples described in Fig. 1D were used for these analyses. The numbers above the lanes represent individual biological replicates.

We next examined the proliferative characteristics of HepYF-M13 and HepYF-M14 cell lines. Both cell lines proliferated rapidly with a doubling time of 11.9 and 16.1 h, respectively (Fig. 2A). Analysis of mitogenic signaling pathways showed that HepYF-M13 and HepYF-M14 cells display high levels of phosphorylated (phospho)-ERK1/2 mitogen-activated protein (MAP) kinase, phospho-AKT, phospho-STAT3, c-Myc, β-catenin (CTNNB1) and cyclin D1 (CCND1) compared to those in primary hepatocytes and/or H2.35 cells (Fig. 2B).

### Transcriptomic analysis and classification of HepYF cells as proliferation class HCC

**Fig. 3.**
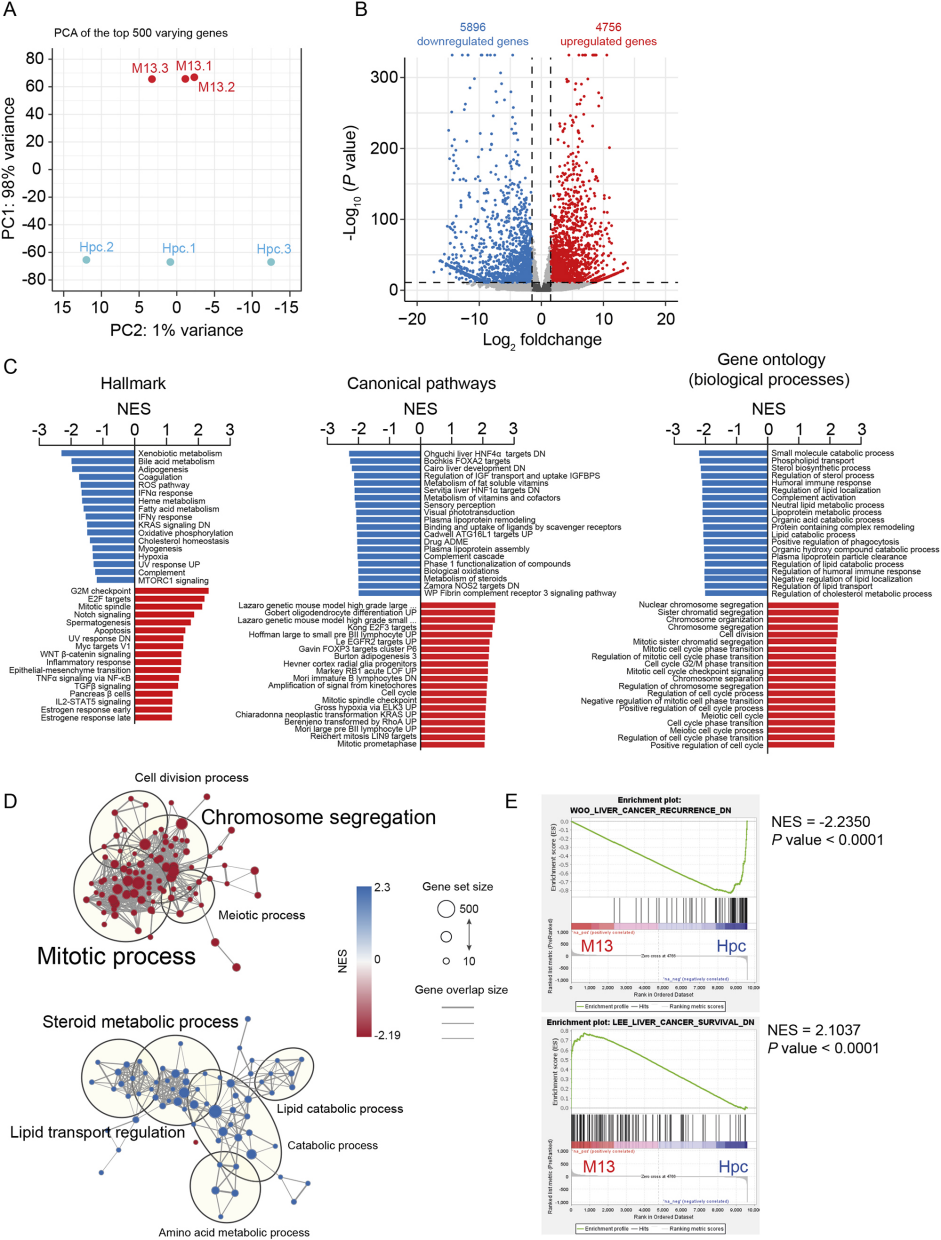
**Transcriptomic analysis of HepYF-M13 cells.** (A) Principal component analysis (PCA) of the top 500 differentially expressed genes in HepYF-M13 cells (red) and primary mouse hepatocytes (blue). Each dot represents a single replicate. (B) Volcano plot of differentially expressed genes between HepYF-M13 cells and primary mouse hepatocytes. Upregulated and downregulated genes with a log_2_(fold change)>1 and *P*<0.05 are represented as red and blue dots, respectively. (C) Gene set enrichment analysis (GSEA) of differentially expressed genes between HepYF-M13 cells and primary mouse hepatocytes. Enriched gene sets are expressed as bar plots. Red bars represent gene sets upregulated in HepYF-M13 cells and blue bars correspond to gene sets upregulated in primary hepatocytes. Left, hallmark gene sets; middle, canonical pathway gene sets; right, Gene Ontology gene sets. The top 20 gene sets are shown. (D) Cell division and cell differentiation networks enriched in HepYF-M13 cells (red circles) and primary hepatocytes (blue circles), respectively. Circles or nodes represent pathways and lines or edges represent genes that are shared among pathways. Only pathways with five or more nodes are shown. Visualization of molecular networks was generated using Cytoscape (https://cytoscape.org) and EnrichmentMap (Reimand et al., 2019; Merico et al., 2011) software. (E) GSEA plots of liver cancer recurrence and liver cancer survival gene sets extracted from the literature (Woo et al., 2008; Lee et al., 2004). ADME, absorption, distribution, metabolism and excretion; DN, downregulation; NES, normalized enrichment score; UP, upregulation.

We analyzed the transcriptome of HepYF-M13 cells by RNA sequencing (RNA-seq) and compared it to that of primary mouse hepatocytes. Principal component analysis showed that replicates of HepYF-M13 cells clustered together and were clearly separated from individual primary mouse hepatocyte cultures (Fig. 3A). We identified 5896 transcripts that were downregulated and 4756 transcripts that were upregulated in HepYF-M13 cells compared to their expression in primary hepatocytes using a twofold cutoff and *P*<0.05 (Fig. 3B). Gene set enrichment analysis (GSEA) of differentially expressed genes using the Molecular Signature database (Liberzon et al., 2011) revealed that HepYF-M13 cells are highly enriched in gene signatures associated with cell cycle control, mitosis, cellular differentiation, metabolism, epithelial-mesenchymal transition (EMT) and cancer (Fig. 3C,D; Tables S2 and S3). In general, genes upregulated in HepYF-M13 cells were mainly associated with cell division, whereas genes downregulated were related to liver metabolic and homeostasis processes. Interestingly, gene sets associated with liver cancer recurrence and survival were significantly enriched in HepYF-M13 cells, suggesting that these cells model advanced HCC, such as those with cholangiocytic marker expression and combined HCC–cholangiocarcinoma (Fig. 3E).

**Fig. 4.**
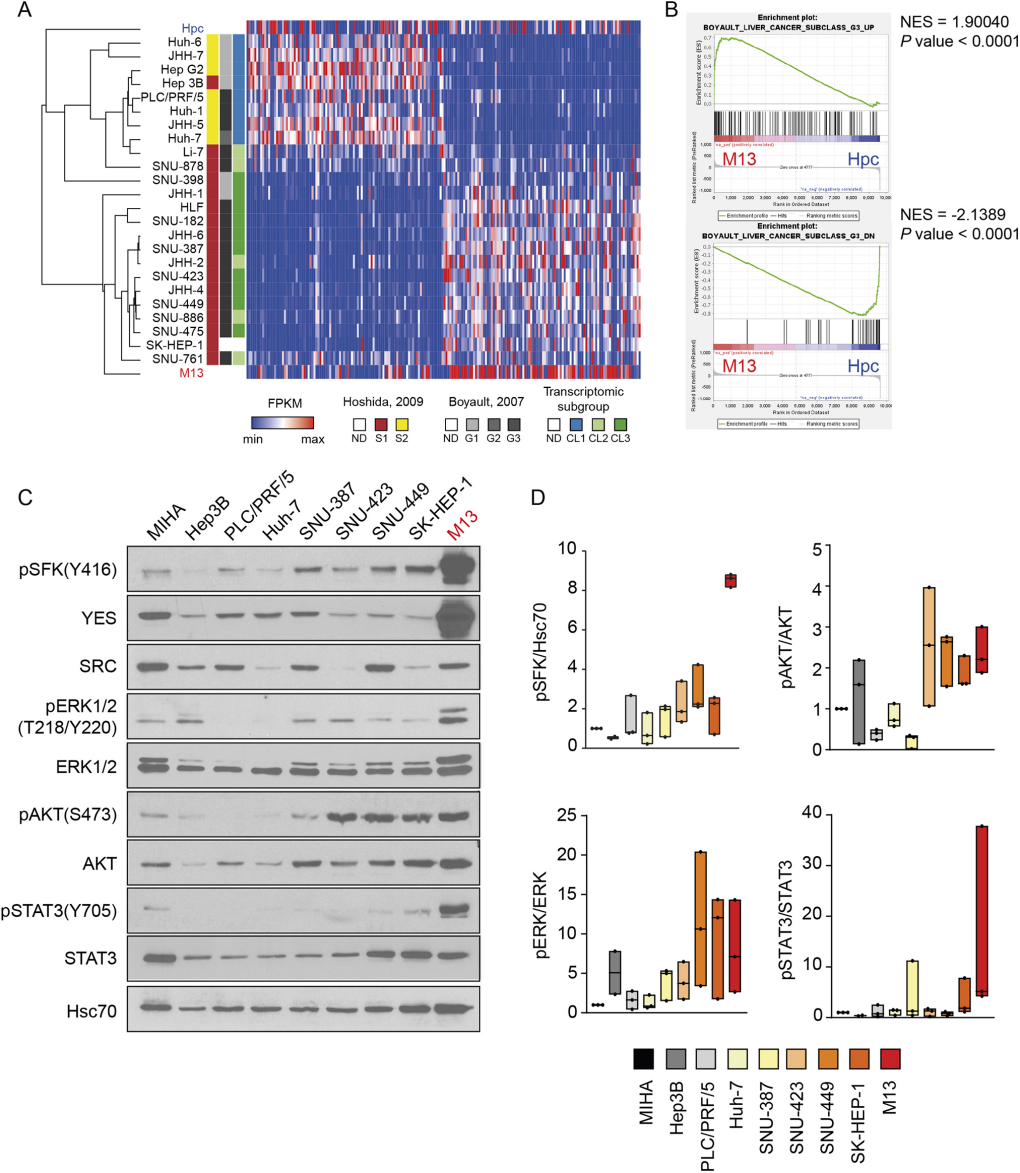
**Classification of HepYF-M13 cells as proliferation class HCC.** (A) Unsupervised hierarchical clustering of HepYF-M13 cells, primary mouse hepatocytes (Hpc) and human HCC cell lines. Transcriptional subgroups of primary HCC tumors and human HCC cell lines are labeled on the left of the heatmap. FPKM, fragments per kilobase of transcript per million mapped reads; ND, not defined. (B) GSEA plots of Boyault's G3 subgroup gene set (Boyault et al., 2007). NES, normalized enrichment score. (C) Western blot analysis of oncogenic signaling pathways in the immortalized human hepatocyte cell line MIHA, indicated human HCC cell lines and HepYF-M13 cells. (D) Quantification of immunoblotting data. Results are presented as the ratio of activating phosphorylation of the indicated signaling proteins over total protein levels (*n*=3). Total protein levels were normalized to Hsc70 abundance. Floating bars display minimal and maximal values with the horizontal line representing the median.

We next compared the transcriptome of HepYF-M13 cells to that of human HCC cell lines to determine whether the mouse HepYF-M13 model recapitulates the gene expression profile of HCC subgroups. In a recent study, Caruso et al. (2019) analyzed the transcriptomic profile of 33 human liver cancer cell lines, resulting in their classification into three subgroups termed CL1 to CL3. By unsupervised clustering analysis, we showed that the global gene expression profile of HepYF-M13 cells was most similar to that of the CL3 subgroup, which defines the less differentiated, proliferative and invasive cell lines expressing high levels of stemness and EMT markers (Fig. 4A). HepYF-M13 cells clustered with HCC cell lines representative of proliferation class HCC tumors of the aggressive G3 subgroup defined by Boyault et al. (2007) and of the Hoshida S1 subgroup (Hoshida et al., 2009) (Fig. 4A). GSEA confirmed that Boyault's G3 subgroup genes were significantly enriched in HepYF-M13 cells (Fig. 4B). These analyses indicate that HepYF-M13 cells are representative of the most aggressive proliferation class HCC.

To further classify HepYF-M13 cells at the molecular level, we measured and compared the level of activation of mitogenic signaling pathways in HepYF-M13 cells and human HCC cell lines representative of the different transcriptomic subgroups. Consistent with the results of Fig. 2B, hyperactivation of SFK, ERK1/2 MAP kinase, AKT and STAT3 pathways was observed in HepYF-M13 cells compared to the immortalized human hepatocyte cell line MIHA (Fig. 4C,D). Notably, a similar activation profile was observed in SK-HEP-1, SNU-449, SNU-423 and SNU-387 cell lines, which are representative of CL3 and G3 subgroups, whereas the cell lines Huh-7, PLC/PRF/5 and Hep3B, representative of the CL1 and G1/G2 subgroups, displayed lower steady-state mitogenic signaling activity (Fig. 4C,D). These results reinforce the conclusion that HepYF-M13 cells are a representative model of advanced proliferation class HCC.

### HepYF cells form ectopic and orthotopic liver tumors in syngeneic hosts

**Fig. 5.**
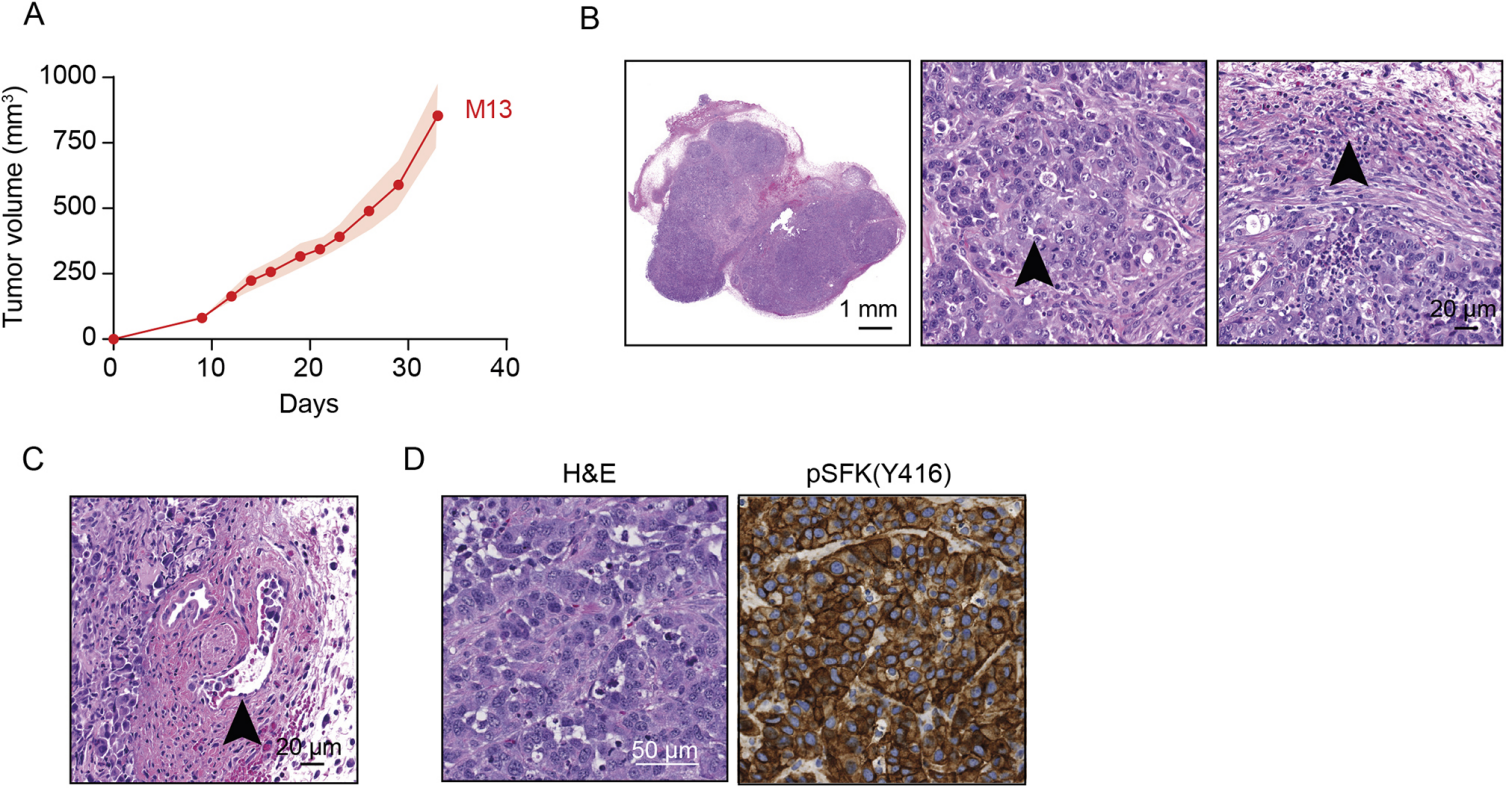
**HepYF-13 cells form ectopic tumors in syngeneic hosts.** (A) Growth curve of HepYF-M13 tumors. HepYF-M13 cells (2×10^6^) were injected subcutaneously in the flank of C57BL/6J mice (*n*=8). Data are mean±s.e.m. (B) Histological features of ectopically implanted tumors in a low-power view (left image), and showing large poorly differentiated cells (arrowhead, middle image) and a brisk immune infiltrate at the tumor edge (arrowhead, right image). (C) Representative photomicrograph of the propensity of HepYF-M13 cells for intravascular invasion, here within a vein (arrowhead). (D) Representative Hematoxylin and Eosin (H&E) staining and immunohistochemistry (IHC) staining of phospho-SFK (Y416) in ectopically implanted tumors. Images are representative of samples from at least three mice.

To evaluate the tumorigenic properties of HepYF-M13 cells, we inoculated syngeneic C57BL/6J mice with 2×10^6^ cells injected subcutaneously in the flank. All mice developed visible tumors by day 7 post injection. The tumors showed a relatively fast growth rate, reaching a volume of 500 mm^3^ by day 26 (Fig. 5A). HepYF-M13 ectopic tumors showed a solid and nested morphology of poorly differentiated cells associated with a significant mononuclear and polymorphonuclear immune infiltrate at the periphery (Fig. 5B). The tumors had multiple areas of necrosis and a mitotic index of up to 27 mitoses per 2 mm^2^ in hotspots. The tumor cells were mostly distributed throughout the subcutaneous tissue, with some areas showing lymphovascular invasion (LVI) at the periphery of the tumor bulk (Fig. 5C). As expected, tumor cells showed uniform intense membrane phospho-SFK immunoreactivity (Fig. 5D).

**Fig. 6.**
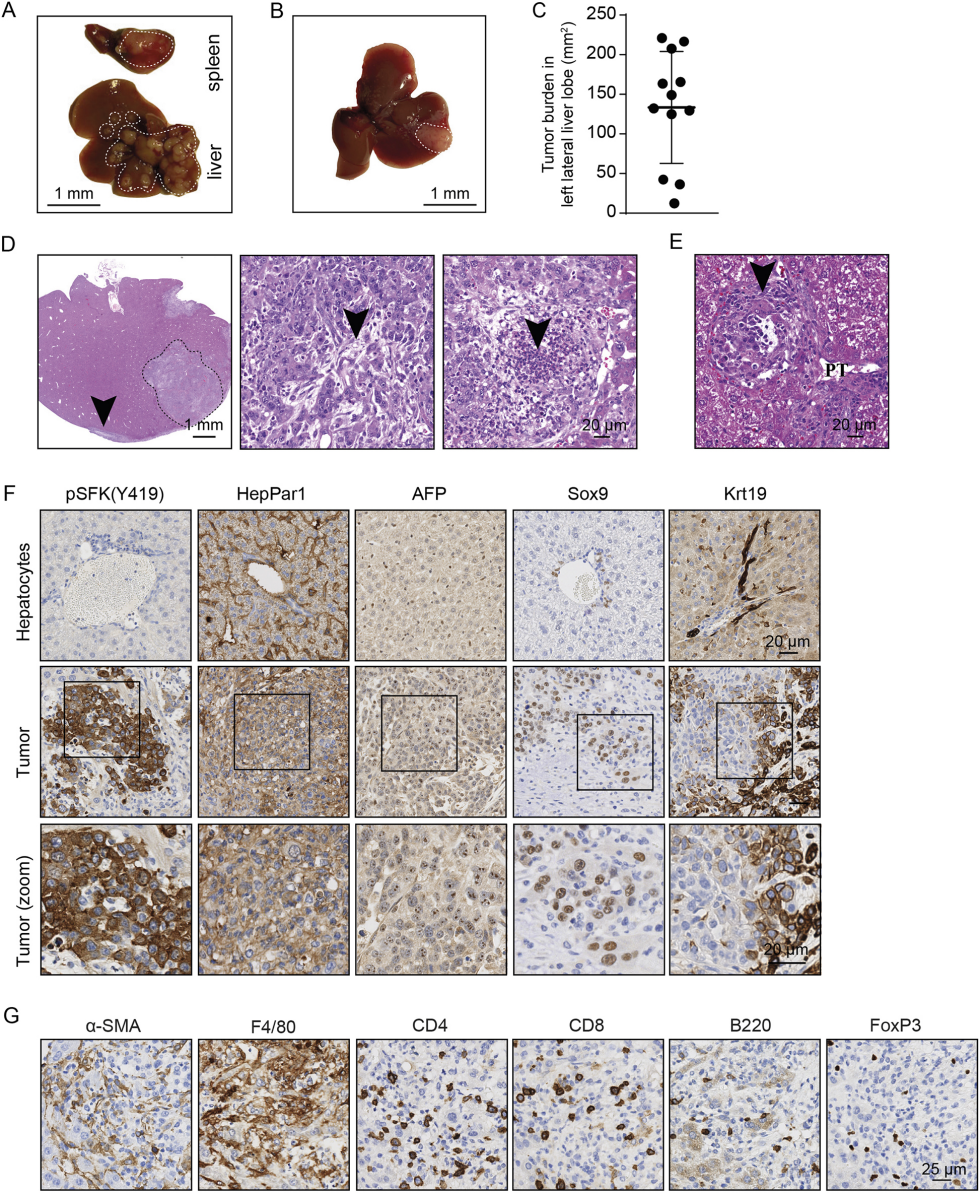
**Orthotopic inoculation of HepYF-13 cells leads to the formation of liver tumors in syngeneic hosts.** (A) HepYF-M13 cells were injected in the spleen parenchyma of C57BL/6J mice (*n*=3). Representative images of liver and spleen at sacrifice 21 days post inoculation are shown. Dashed lines demarcate the tumor from the adjacent normal tissue. (B,C) HepYF-M13 cells were injected in the liver parenchyma of C57BL/6J mice (*n*=12). A representative image of a single liver tumor nodule 8 days post inoculation is shown (dashed line, B). Quantification of area of tumor nodules measured on H&E-stained liver sections (C). Data are mean±s.d. (D) Representative photomicrographs of liver sections stained with H&E from C57BL/6J mice orthotopically inoculated with HepYF-M13 cells in the liver parenchyma. Left image, low-power view showing a main tumor nodule at the site of injection (dashed line) and subcapsular extension (arrowhead). At high power, tumor cells show a fibroblastic reaction (arrowhead, middle image), and the formation of necrotic microabscesses (arrowhead, right image). (E) Photomicrograph showing the presence of tumor deposits (arrowhead) at remote locations away from the main nodule within a hepatic portal tract (PT). (F) Expression of phospho-SFK (Y416), the hepatocyte markers HepPar1 and AFP, the stem cell marker Sox9, and the cholangiocyte marker Krt19 in intrahepatic tumors. (G) Representative IHC staining for α-smooth muscle actin (αSMA), F4/80, CD4, CD8, B220 and FoxP3 in sections from intrahepatic tumors. Images are representative of samples from 12 mice (H&E) and three mice (IHC).

We evaluated whether the newly derived HCC cell lines could be used as orthotopic HCC models, which more accurately reflect the native tumor microenvironment, vascularization and interaction with immune cells. As a first intrasplenic injection model, we injected HepYF-M13 cells into the spleen parenchyma of C57BL/6J mice and sacrificed the animals after 21 days. Liver tumor engraftment was observed in two out of three mice, and tumors were also detected in the spleen of all mice (Fig. 6A). Macroscopic analysis showed that the tumors were multinodular, with varying sizes and numbers. This complicates the measurement of tumor burden and is not practical for preclinical studies. As a second model, we evaluated the intrahepatic injection of tumor cells. Inoculation of HepYF-M13 cells under the liver capsule led to the formation of liver tumors in 100% (*n*=12) of mice. All mice developed a single dominant tumor nodule of variable size (Fig. 6B,C). The tumors showed a similar poorly differentiated morphology with a propensity for spindle shapes (Fig. 6D). A significant fibroblastic reaction was present in all tumors, and the immune infiltrate was predominantly neutrophilic with clusters of necrosis and microabscesses (Fig. 6D). The mitotic index was estimated at up to 24 mitoses per 2 mm^2^ in hotspots. Similar to the vascular invasive capacity of the subcutaneous model, the orthotopic model had multiple microscopic extension of the tumor away from the dominant nodule, mostly located in portal tracts (Fig. 6E). Immunohistochemistry (IHC) analysis showed that HepYF-M13 tumors exhibited high phospho-SFK staining (Fig. 6F). The orthotopic tumors were also positive for the hepatocellular markers HepPar1 and α-fetoprotein (AFP), whereas they overexpressed the stemness marker Sox9. Consistent with gene expression data, staining with Krt19 highlighted a subset of tumor cells with variable expression of Krt19 (Fig. 6F).

We performed a basic immune phenotyping of the tumor microenvironment by IHC to describe the immune and fibroblastic composition of HepYF-M13 tumors (Fig. 6G). Fibroblasts in the tumor showed an activated phenotype with α-smooth muscle actin (α-SMA or ACTA2) expression and stained 6-16% of the tumor surface area. The most significant immune population was macrophages (positive for F4/80 or ADGRE1), representing 31-41% of all cells within the tumoral area. CD4-, CD8 (or CD8A)-, and B220 (encoded by *Ptprc*)-expressing T and B lymphocytes represented 4.4-10.3% of all cells, with CD4^+^ lymphocytes representing 1.4-4.8%, CD8^+^ lymphocytes representing 1.7-4.0% and B220^+^ lymphocytes representing 0.7-1.9% of cells. A minority of cells expressing FoxP3 was identified in 0.5-0.7% of all cells within the tumors.

We compared the ectopic and intrahepatic HepYF-M13 tumors to the original liver tumors resulting from hydrodynamic tail vein injection of *YES1* Y537F, from which HepYF-M13 cells are derived. The original genetically engineered tumors reiterated features found in well-differentiated and moderately differentiated human HCC. The hepatocyte cell plate ranged between two and four cells in thickness, and the cells had preserved vacuoles (Fig. S1). The original tumors were positive for AFP and HepPar1 similarly to transplanted HepYF-M13 tumors but showed reduced Sox9 and no significant Krt19 expression in tumor cells (Fig. S1). As the HepYF-M13 cell line was established from these original tumors, this further supports the acquisition of Sox9 and Krt19 expression between the two timepoints, reminiscent of the increased aggressiveness in human HCC (Govaere et al., 2014; Richtig et al., 2017).

The murine HCC cell line Hepa 1-6, originally isolated from a spontaneous liver tumor in C57L/J mice (Darlington, 1987), is the most commonly used syngeneic orthotopic HCC model. To compare the HepYF-M13 orthotopic model to this established HCC model, we injected the same number of Hepa 1-6 cells into the liver of 6 C57BL/6J mice. All six mice developed a tumor mass, but the size of the tumors was markedly smaller than that seen for the HepYF-M13 model (Fig. S2A,B). The Hepa 1-6 tumors showed a trabecular pattern with preserved sinusoidal structures. Microabcesses were observed similarly to the HepYF-M13 tumors (Fig. S2C). Distinct from the HepYF-M13 model, we did not observe a fibroblastic reaction but, instead, noticed chronic type inflammation at the tumor–hepatocyte interface (Fig. S2D).

### HepYF orthotopic tumors develop intrahepatic and extrahepatic metastases

Patients with early HCC recurrence because of intrahepatic metastasis or patients with extrahepatic metastases from advanced HCC have a poor prognosis (Natsuizaka et al., 2005; Yang et al., 2017). There is a need to develop novel, relevant animal models of HCC intrahepatic and extrahepatic metastases to study the molecular pathogenesis of metastatic HCC and evaluate the efficacy of systemic therapies in preclinical studies. The classical sequence of events from localized to metastatic disease observed in humans is the initial growth of the primary tumor, followed by LVI, and eventually lymph node and distant metastases (Amin et al., 2017). This sequence follows the anatomic and physiologic lymphatic and venous drainage sequences of the liver. There are several models that have demonstrated direct seeding of tumors into the lung, by direct tail vein injection of tumor cells into often immunosuppressed mice, as cancer is deposited artificially into organs. However, very few, if any, models reiterate the complete sequence of HCC intrahepatic and extrahepatic localization: primary tumor, LVI, lymph node metastases and distant metastases.

**Fig. 7.**
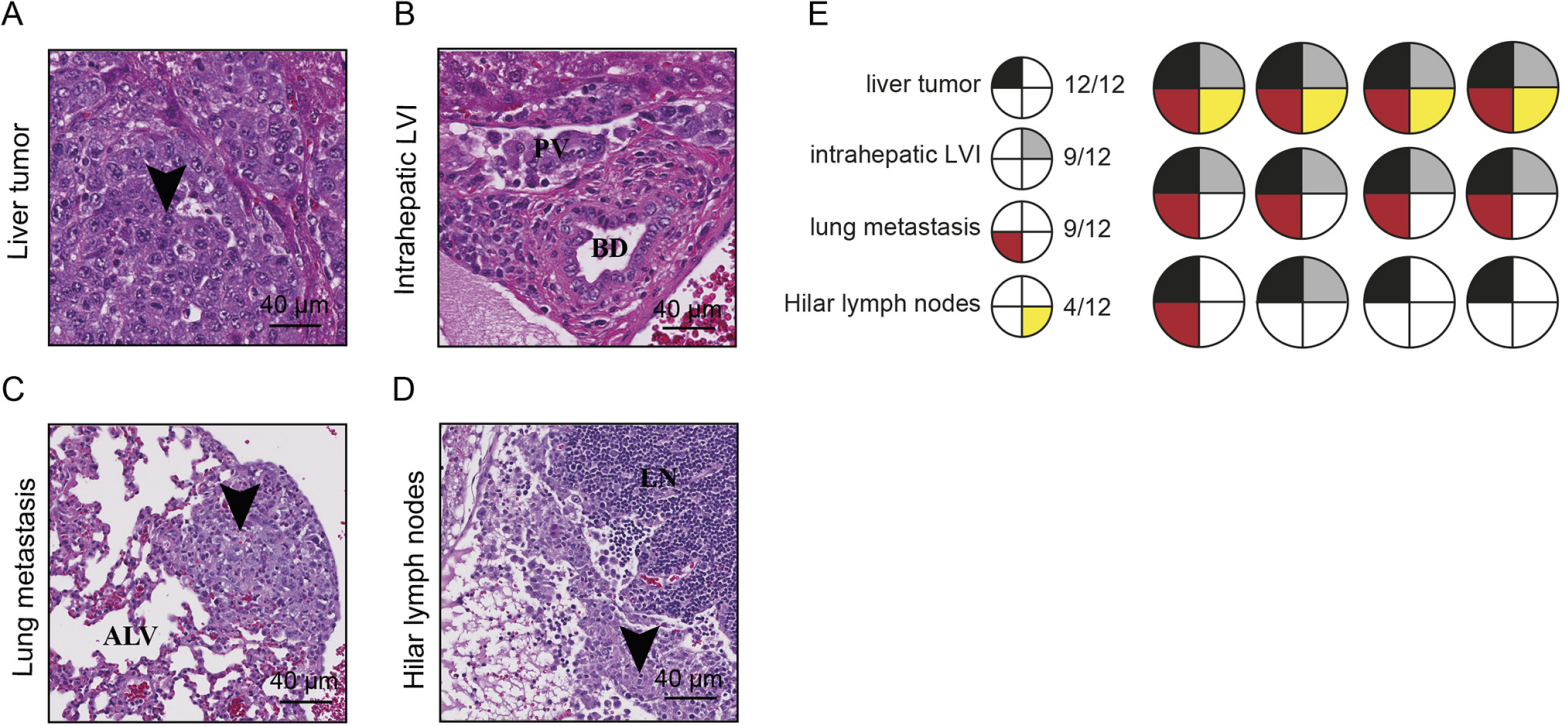
**Tumor implantation and dissemination following intrahepatic inoculation of HepYF-M13 cells.** (A) Tumor cells at the site of inoculation (arrowhead). (B) Vascular invasion within a portal venule (PV) in the liver, away from the main tumor. BD, bile duct; LVI, lymphovascular invasion. (C) Metastatic dissemination of tumor cells in the lung parenchyma (arrowhead), within alveolae (ALV). (D) Metastatic dissemination of tumor cells within a hilar (pulmonary) lymph node (arrowhead) in the lymph node tissue (LN). Scale bars: 40 µm. (E) Schematic representation of tumor localization in mice (*n*=12) orthotopically inoculated with HepYF-M13 cells.

To further explore the vascular invasive properties of the HepYF-M13 cell line, we transplanted the cells in the liver parenchyma of 12 C57BL/6J mice and euthanized them after 8 days. Detailed histological examination revealed the capacity of HepYF-M13 cells for intrahepatic LVI, pulmonary macro- and micro-metastases, and dissemination in the pulmonary hilar lymph nodes (Fig. 7A-D). The anatomical sequence of hepatic tumor, intrahepatic LVI, pulmonary metastases and, finally, hilar lymph nodes was mostly conserved, as LVI was nearly always visualized prior to distant metastases (Fig. 7E). Overall, our HepYF-M13 orthotopic model achieved significant implantation and dissemination properties, with 100% intrahepatic tumors, 75% intrahepatic LVI, 75% lung metastases and 33% hilar lymph node metastases. These observations are in line with the high grade of HepYF-M13 orthotopic tumors and with the proliferation-, EMT- and poor outcome-associated gene signatures of the cells (Fig. 3C-E).

For comparison purposes, we also analyzed the disseminative properties of the Hepa 1-6 orthotopic model. After fully submitting the six Hepa 1-6 liver tumors generated by intrahepatic injection (described in Fig. S2), none showed LVI or portal dissemination. We also fully submitted pulmonary and hilar structures for histological examination and no cancer cells were detected at this time point. These findings contrast with the highly disseminated nature of the HepYF-M13 cell line.

### HepYF tumors are responsive to small-molecule kinase inhibitors and immune checkpoint blockade

**Fig. 8.**
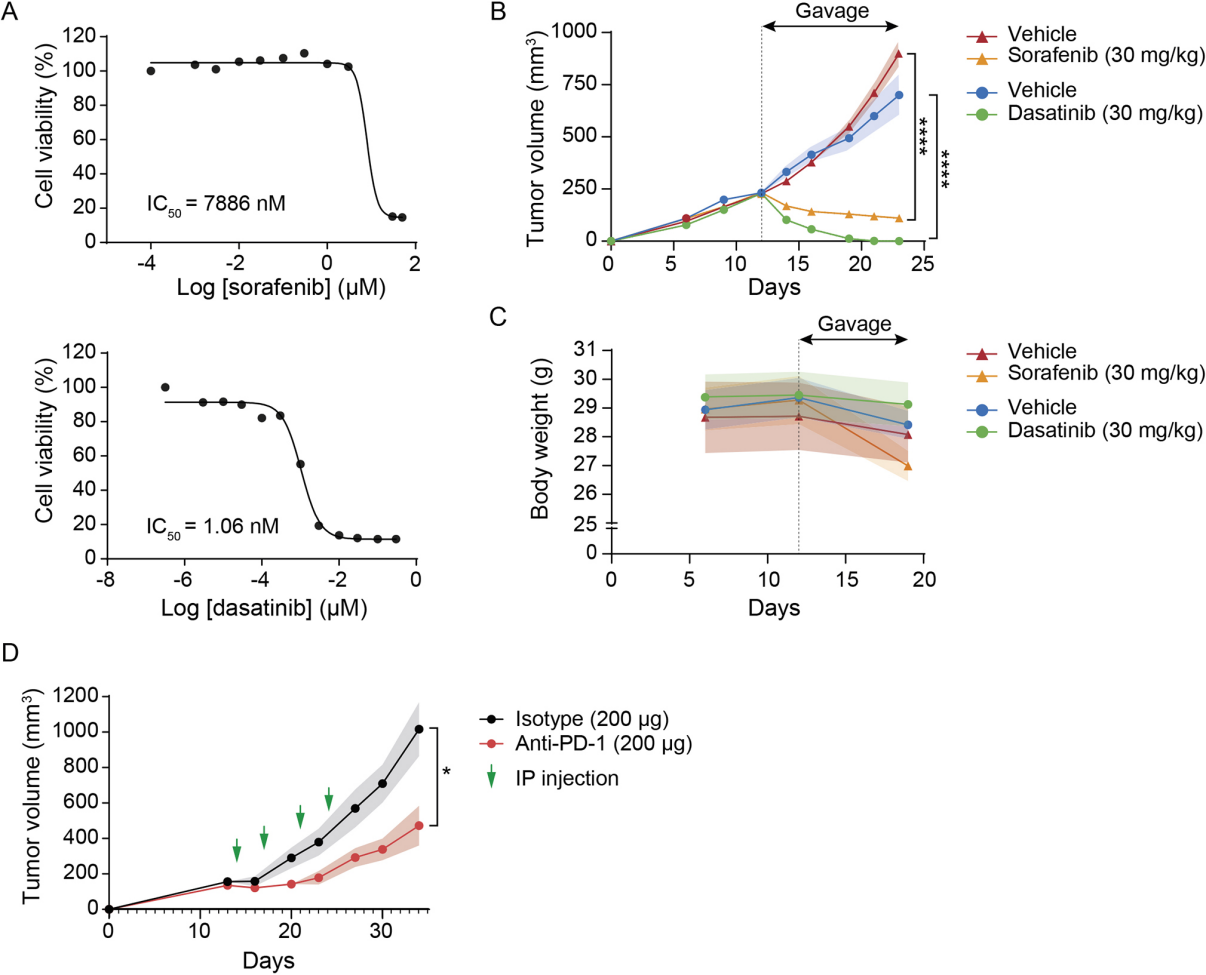
**Effect of small-molecule kinase inhibitors and immune checkpoint blockade on the growth of HepYF-13 tumors.** (A) *In vitro* cell proliferation assays. Dose-response curves of sorafenib and dasatinib on the proliferation of HepYF-M13 cells. IC_50_ values are indicated and represent the mean of three experiments. (B,C) *In vivo* efficacy study of kinase inhibitors. HepYF-M13 cells were injected subcutaneously in C57BL/6J mice. Once tumors reached a volume of 200-250 mm^3^, the mice (*n*=8 mice/group) were randomized and treated with vehicle, sorafenib (30 mg/kg) or dasatinib (30 mg/kg) administered daily by oral gavage for 2 weeks. Tumor growth was monitored bi-weekly (B) and body weight was recorded every week (C). Data are mean±s.e.m. ****P<0.001 by unpaired two-tailed Student's *t*-test. (D) *In vivo* efficacy study of anti-PD-1 antibody. Once HepYF-M13 tumors reached a volume of 150-200 mm^3^, the mice (*n*=5 mice/group) were randomized and treated with isotype IgG2a control or anti-PD-1 (200 µg/mouse) administered bi-weekly by intraperitoneal (IP) injection for 2 weeks. Tumor growth was monitored daily and the mice were sacrificed 10 days after the last dose. Data are mean±s.e.m. *P<0.05 by unpaired two-tailed Student's *t*-test.

We evaluated the relevance of the novel syngeneic HCC models for preclinical testing of small-molecule-targeted therapies and immune checkpoint inhibitors. We first tested the effect of the first-line systemic agent sorafenib on the *in vitro* proliferation of HepYF-M13 cells. Treatment with sorafenib inhibited the proliferation of HepYF-M13 cells with weak potency (Fig. 8A), similar to its effect on human HCC cell lines (Liu et al., 2006). As expected, the non-selective SFK inhibitor dasatinib potently inhibited cell proliferation at single-digit nanomolar potency, reflecting the oncogenic addiction of HepYF-M13 cells to YES signaling (Fig. 8A).

We next tested the efficacy of the two drugs *in vivo*. For these studies, C57BL/6J mice were inoculated with HepYF-M13 cells injected subcutaneously in the right flank. Once tumors reached an average volume of 150-200 mm^3^, the mice were randomly allocated to the different study arms and treated daily for a period of 14 days. Treatment with sorafenib at 30 mg/kg markedly reduced the growth rate of HepYF-M13 tumors and induced tumor regression in all individual mice (Fig. 8B). Treatment with dasatinib (30 mg/kg) elicited a stronger response, also leading to complete tumor regression in all animals. No significant change in body weight was observed throughout the study (Fig. 8C). To determine whether HepYF-M13 tumors are responsive to immune checkpoint blockade, syngeneic C57BL/6J mice were similarly inoculated with HepYF-M13 cells and treated with 200 µg antibody against PD-1 (or PDCD1) injected bi-weekly for a period of 2 weeks. The mice were sacrificed 10 days later. Treatment with anti-PD-1 resulted in a marked reduction of tumor growth in this model (Fig. 8D). These results provide proof of concept of the value of the HepYF-M13 cell line model for the preclinical evaluation of new HCC therapeutic modalities.

## DISCUSSION

HCC is a complex disease associated with multiple genomic and epigenomic alterations, and dysregulation of various signaling pathways (Llovet et al., 2021). Integrative genomic and pathway analysis have led to the classification of HCC into two major classes, a proliferation and a non-proliferation class, each accounting for approximately 50% of cases (Llovet et al., 2015; Zucman-Rossi et al., 2015). Each class integrates different molecular subclasses defined through multiple genomic studies. Recently, we identified a previously uncharacterized YES-driven oncogenic signaling pathway in HCC (Guégan et al., 2022). We showed that YES activation is both necessary and sufficient for liver tumor formation in the mouse. The clinical relevance of these findings is suggested by the observation that SFKs are hyperactivated in ∼25% of human HCC, associated with increased tumor burden. Although SFK genes are rarely mutated in human cancers, several studies have documented the increased expression and/or activation of SFKs in various solid cancers (Gelman, 2011; Kim et al., 2009; Martellucci et al., 2020). However, there is no relevant preclinical animal model of SFK-driven cancer.

The objective of this study was to develop and characterize a transplantation mouse model of YES-induced HCC to study the liver oncogenic network of this tyrosine kinase and evaluate the efficacy of novel targeted therapies. To this end, we established cell lines from primary liver tumors surgically resected from transgenic mice hydrodynamically injected with activated *YES1* Y537F. Neoplastic HepYF cells isolated from these primary liver tumors formed new tumors when ectopically transplanted into NSG mice, but were rejected by immunocompetent C57BL/6J mice. However, upon secondary transplantation, HepYF cell lines successfully engrafted into syngeneic C57BL/6J mice. Pathological, gene expression and protein studies confirmed that HepYF cells model liver cancers with significant stemness and proliferative properties. HepYF-M13 tumors were poorly differentiated and were positive for HepPar1 and AFP, suggestive of a hepatocellular lineage. Furthermore, Krt19 IHC highlighted a double population of cells with strong expression and adjacent zones with reduced expression, absent in the original transgenic *YES1* Y537F liver cancer model from which HepYF-M13 cells were derived. This parallels the acquisition of an aggressive phenotype seen in patients with HCC (Govaere et al., 2014). The heterogeneous acquisition of a Krt19 phenotype by this model makes it a suitable tool to study the acquisition of aggressive traits during HCC progression. Collectively, these findings identify HepYF-M13 tumors as HCCs with aberrant expression of cholangiocellular markers, grouped within the proliferation molecular subclass (Brunt et al., 2018; Llovet et al., 2016; Murugesan et al., 2021; Stavraka et al., 2019). Our transcriptomic data validated this idea, as HepYF cells exhibited significant similarities in expression profile to that of proliferation class human HCCs. These tumors are highly aggressive and, similarly to our orthotopic model findings, prone to metastases.

HepYF cell lines proliferated rapidly and were characterized by the hyperactivation of multiple oncogenic signaling pathways, such as the ERK1/2 MAP kinase, the phosphatidylinositol 3-kinase/AKT and the STAT3 pathways, and by the increased expression of c-Myc, β-catenin and the cell cycle effector cyclin D1. As expected, the cells showed a strong activation of SFK signaling, evidenced by the augmented phosphorylation of FAK on Y925. Consistent with these results, transcriptomic profiling of the HepYF-M13 cell line revealed an enrichment of multiple gene sets associated with cell proliferation and mitosis. Unsupervised hierarchical clustering analysis of HepYF-M13 cells and human HCC cell lines showed that HepYF-M13 cells clustered mainly with mesenchymal-like human HCC cell lines of the CL3 transcriptomic subgroup, which includes less differentiated, highly proliferative and invasive liver cancer cells that express high levels of stemness and EMT markers (Caruso et al., 2019). Consistent with this classification, HepYF-M13 cells also displayed gene signatures associated with liver cancer recurrence and poor outcome. Thus, HepYF-M13 represents a valuable model of proliferation class HCC and, more specifically, of the most aggressive CL3 subgroup.

The HepYF model presents several advantages for the study of liver cancer. First, tumor growth is rapid, predictable and reproducible. This is important for the preclinical evaluation of novel therapies. Second, it is versatile and tumors can be established by either subcutaneous, intrasplenic or orthotopic intrahepatic inoculation of HepYF cells. Subcutaneous implantation is relatively easy to perform, allows direct monitoring of tumor progression without an imaging facility, and is well suited for drug efficacy studies. However, this model does not take the hepatic tumor microenvironment into consideration. Intrasplenic injection can be useful as a model of intrahepatic metastasis, but it requires surgical expertise and gives rise to spleen tumors and to diffuse multinodular liver tumors of variable sizes. Intrahepatic injection more accurately reflects the native tumor microenvironment and leads to rapidly growing single tumor lesions, but it is also technically more challenging. Thus, depending on the specific question being asked, different experimental approaches can be used with this model. Third, HepYF tumors can be established in syngeneic hosts, allowing the study of liver antitumor immune responses and the evaluation of immunotherapies. Immune checkpoint inhibitors are now approved as a second-line treatment for advanced HCC, and the combination of atezolizumab and bevacizumab was recently approved in the first-line setting (Llovet et al., 2022). Many new combination regiments with immunotherapies are currently being developed that need to be tested in relevant syngeneic models of liver cancer. Finally, our model replicates the most aggressive form of human liver cancer, which requires systemic therapy. Indeed, most murine models of HCC generate intrahepatic tumors that lead to hepatic failure prior to distant dissemination (Brown et al., 2018). This is equivalent to cirrhotic patients awaiting liver transplants, who die of liver failure due to the intrahepatic cancer exceeding the Milan criteria for transplantation. However, systemic therapies are mostly used in highly metastatic disease, which the HepYF-M13 orthotopic model replicates in an efficient manner. Importantly, we observed similar disseminative properties as in humans, with a propensity for pulmonary localization (Katyal et al., 2000).

Although the distinction between artificial seeding at the time of injection versus a more organic implantation–dissemination event remains elusive, there are significant physiological findings that suggest a metastatic process. First, LVI was unequivocally identified 8 days following intrahepatic injection. These detached clusters of cells were located inside vascular channels, unattached to the wall, strongly indicative of continuous clusters of HepYF-M13 cells being released into the venous circulation at day 8. Second, thoracic hilar lymph nodes were in a physiologically distinct compartment from the venous–arterial vascularization. If we believe that this was an artificial seeding event, this entails that HepYF-M13 cells penetrated the first layer of lung capillaries, then simultaneously transgressed the lymphatic endothelial layer to seed hilar lymph nodes at day 0. Altogether, there is evidence that the HepYF-M13 model can continuously release cancer clusters into the vascular compartment and can penetrate vascular barriers with ease, akin to metastases. However, we do not exclude the possibility that microscopic or macroscopic metastases may result from tumor cells artificially entering the circulation at the time of intrahepatic injection. The distinction between artificial seeding and metastases, or even both simultaneously, will remain challenging to determine.

As a proof of concept of the value of HepYF models for the evaluation of novel HCC therapies, we inoculated C57BL/6J mice with HepYF-M13 cells and, once tumors were established, treated the mice with the small-molecule kinase inhibitors dasatinib and sorafenib, or an immune checkpoint inhibitor. Oral administration of dasatinib induced a rapid and robust regression of tumor growth, consistent with the oncogenic addiction of HepYF-M13 cells to activated YES. To further test the translational potential of the HepYF-13 model, we treated a group of mice with the multi-kinase inhibitor sorafenib, a first-line HCC therapy. Treatment with sorafenib also induced a regression of HepYF-M13 tumors, albeit with slightly lower efficacy than that for dasatinib. Notably, anti-PD-1 immunotherapy also resulted in a marked reduction of tumor growth. These pharmacological studies validate the translational value of HepYF models for the preclinical investigation of novel HCC therapies. The ability to perform efficacy studies in immunocompetent hosts represents a major advantage, given that many current and investigational HCC therapeutics target the immune system.

## MATERIALS AND METHODS

### Reagents, plasmids and antibodies

Dasatinib (D-3307) and sorafenib (S-8599) were purchased from LC Laboratories. *InVivo*MAb rat IgG2a isotype control (clone 2A3, BE0089) and anti-mouse PD-1 (clone RMPI-14, BE0146) antibodies were purchased from Bio X Cell. The plasmids pT3-EF1α-YES Y537F and pCMV-T7-SB100 have been described previously (Guégan et al., 2022). Commercial antibodies for western blotting were obtained from the following suppliers: anti-YES (610375; 1/1000) from BD Biosciences-Pharmingen; anti-SRC (32G6; 1/1000), anti-FAK (3285; 1/1000), anti-phospho-FAK (Y925) (3284; 1/1000), anti-ERK1/2 (137F5; 1/1000), anti-phospho-SFK (Y416) (6943; 1/1000), anti-phospho-ERK (T202/Y204) (E10; 1/1000), anti-phospho-AKT (S473) (D9E; 1/1000), anti-AKT (9272; 1/1000), anti-phospho-STAT3 (Y705) (D3A7; 1/1000), anti-STAT3 (124H6; 1/1000) and anti-YAP/TAZ (D24E4; 1/1000) from Cell Signaling Technology; anti-Hsc70 (B-6; 1/2000) and anti-cyclin D1 (R-124; 1/500) from Santa Cruz Biotechnology; anti-c-Myc (Y69; 1/1000), anti-Sox9 (82630; 1/1000) and anti-Hnf4a (ab181604; 1/500) from Abcam; anti-β-catenin (2337-1; 1/2000) from Epitomics; and anti-glutamine synthetase (610517; 1/500) from Pharmingen. Antibodies for IHC were obtained from the following suppliers: anti-phospho-SFK (Y416) (2101; 1/100 for 60 min), anti-Sox9 (82630; 1/150 for 60 min), anti-α-SMA (19245; 1/200 for 30 min), anti-CD8 (98941; 1/200 for 30 min) and anti-FoxP3 (12653; 1/400 for 30 min) from Cell Signaling Technology; HepPar1 (ab190706; 1/2000 for 15 min), anti-Krt19 (ab52625; 1/400 for 30 min) and anti-CD4 (ab183685; 1/1000 for 30 min) from Abcam; anti-AFP (14550-1-AP; 1/100 for 60 min) from Proteintech; anti-B220 (14-0452-82; 1/200 for 15 min) from Invitrogen; and anti-F4/80 (MCA497R; 1/100 for 30 min) from Bio-Rad.

### Mice

C57BL/6J and NSG mice were obtained from The Jackson Laboratory. All mice were bred under standard conditions at the Institute for Research in Immunology and Cancer. Mice were housed under specific pathogen-free conditions in filter-topped isolator cages with access to food and water *ad libitum*. Animals were handled in strict accordance with good animal practice as defined by the relevant local animal welfare bodies, and all experiments were approved by the Canadian Council on Animal Care (CCAC).

### Generation of *YES1* Y537F transgenic mice

Hydrodynamic transfection of hepatocytes was performed as previously described (Kovacsics and Raper, 2014). The lateral tail veins of C57BL/6J mice (average weight of 22 g) were injected with the plasmids pT3-EF1α-YES Y537F (10 µg/ml) and pCMV-T7-SB100 (1 µg/ml), encoding the Sleeping Beauty transposase, diluted in 0.9% NaCl. The volume of injection corresponded to 10% of the mouse body weight and the injection was performed within 5-7 s. Liver tumors were processed as detailed below.

### Isolation of HepYF cell lines and cell culture

Five to 7 months after hydrodynamic gene delivery of *YES1* Y537F, liver tumors were micro-dissected, and the tissue was minced with a scalpel blade and digested with 1% trypsin (Gibco)/1% collagenase (Roche) for 30 min at 37°C. The dissociated cells were plated in 10-cm plates and cultured in Dulbecco's modified Eagle medium (DMEM; Thermo Fisher Scientific) supplemented with 10% fetal bovine serum (Wisent) and antibiotics. Cell lines were established after passaging the cells four to five times over a period of 4 to 6 weeks. The original cell lines (4×10^6^ cells) were then injected subcutaneously into the flank of NSG and C57BL/6J mice. Allograft tumors from NSG mice were excised, and tumor cells were dissociated and expanded for multiple passages as described above. Newly established cell lines were re-injected in NSG and C57BL/6J mice. HepYF cells were maintained in DMEM supplemented with 10% fetal bovine serum and antibiotics. Hepa 1-6 cells were obtained from the American Type Culture Collection and cultured in DMEM supplemented with 10% fetal bovine serum and antibiotics. The cells were routinely tested for mycoplasma contamination. Mouse HCC cell lines are available from the Meloche laboratory upon request.

### Genotyping and DNA sequencing

Genomic DNA from HepYF-M13 cells was extracted using the Phire Tissue direct PCR Master Mix (Thermo Fisher Scientific) according to the manufacturer's instructions. For DNA sequencing, amplification of the transgenic *YES1* genomic region was performed by PCR using the primers 5′-CTAGTAACAAAGGGCCGAGTGCCATATCC-3′ and 5′-TGGCTGGCAACTAGAAGGCACAGTCGAGGC-3′, followed by Sanger sequencing on an Applied Biosystems 3730 DNA analyzer.

### Isolation of primary hepatocytes

Primary hepatocytes were isolated from the livers of 8- to 12-week-old male C57BL/6J mice by collagenase dissociation. The mice were anesthetized, positioned on a dissection tray, and the inferior vena cava was cannulated and the liver perfused with warm HEPES-buffered saline for 10 min using a peristaltic pump at a flow rate of 3 ml/min to remove blood and circulating cells. Then, the liver was perfused with a solution of 600 µg/ml collagenase (Sigma-Aldrich, C5138) for 4 min to facilitate cell dissociation. The liver was dissected out and the hepatic lobes were torn apart to release hepatocytes in a cell culture plate. The hepatocyte suspension was filtered through a 70 µm cell strainer, washed two times with Hepatocyte Wash Medium (Thermo Fisher Scientific), and filtered one more time through a 40 µm cell strainer in culture medium. Hepatocytes were plated at near confluence in DMEM supplemented with 10% fetal bovine serum, 0.1% bovine serum albumin, antibiotics (penicillin, streptomycin and fungizone) and 100 nM dexamethasone. Cell viability was estimated to be >80% by Trypan Blue exclusion. After adhesion for 1-2 h, hepatocytes were washed twice and maintained in culture for up to 48 h by changing the medium every day.

### Cell lines

SNU-387, SNU-423, SNU-449, PLC/PRF/5, SK-HEP-1, Hep3B and Huh-7 cells were obtained from the American Type Culture Collection. The human immortalized hepatocyte cell line MIHA (Brown et al., 2000) was a gift from Dr Jayanta Roy-Chowdhury (Albert Einstein College of Medicine, NY, USA). The mouse immortalized hepatocyte cell line H2.35 was kindly provided by Dr Jennifer Estall (Institut de recherches cliniques de Montréal, Canada). Cell lines were cultured in DMEM supplemented with 10% fetal bovine serum and antibiotics. The cells were routinely tested for mycoplasma contamination.

### Immunoblot analysis

Cells were washed twice with ice-cold PBS and lysed in 1% Triton X-100 lysis buffer (50 mM Tris-HCl, pH 7.4, 100 mM NaCl, 50 mM sodium fluoride, 5 mM EDTA, 40 mM β-glycerophosphate, 1 mM sodium orthovanadate, 0.1 mM phenylmethylsulfonyl fluoride, 1 μM leupeptin, 1 μM pepstatin A) for 30 min at 4°C. Lysates were clarified by centrifugation at 13,000 ***g*** for 5 min and lysate proteins were subjected to electrophoresis on acrylamide gels. Proteins were electrophoretically transferred to 0.45 µm nitrocellulose membranes (Bio-Rad) in 20% ethanol transfer buffer (25 mM Tris, 192 mM glycine, pH 8.3). The membranes were blocked in Tris-buffered saline containing 5% nonfat dry milk and 0.1% Tween 20 for 1 h at room temperature before incubation overnight at 4°C. After washing four times in Tris-buffered saline containing 0.1% Tween 20, the membranes were incubated for 1 h with horseradish peroxidase-conjugated goat anti-rabbit or anti-mouse IgG (1:10,000, Bio-Rad, 170-6515 and 170-6516) in blocking solution. Immunoreactive bands were visualized by enhanced chemiluminescence (Thermo Fisher Scientific).

### Real-time qPCR

Total RNA was isolated using the RNeasy isolation kit (Qiagen) and converted to cDNA using the Maxima first-strand cDNA synthesis kit with dsDNase (Thermo Fisher Scientific). Gene expression was measured using assays designed for the Universal ProbeLibrary system (Roche) as described previously (Guégan et al., 2022). The cycle threshold (Ct) value for each gene was normalised to the Ct value of *Actb* and *Hprt* or *Gapdh* and *Hprt* and the relative levels of expression were calculated. Primer sequences are listed in Table S4.

### Cell proliferation assay

Cell proliferation was measured by the WST1 assay (Roche) according to the manufacturer's instructions.

### Transcriptomic analysis

Total RNA from C57BL/6J primary hepatocytes (*n*=3) and HepYF-M13 cells (*n*=3) was isolated using the RNeasy purification kit (Qiagen) and the quality was assessed on an Agilent 2100 BioAnalyzer. Libraries (400 ng total RNA) were prepared using the KAPA Hyperprep Stranded mRNA-Seq Kit (KAPA Biosystems). cDNA fragments were ligated to indexed library adapters (Illumina) prior to PCR amplification (ten cycles). Purified libraries were normalized by qPCR using the KAPA Library Quantification Kit (KAPA Biosystems) and diluted to a final concentration of 10 nM. The libraries were pooled and sequenced on an Illumina NextSeq500 sequencer using the Nextseq HighOutput v2 Kit (75 cycles, single end) and the pooled library at 2 pM. Between 21 to 25 million single-end reads were generated per sample. Library preparation and sequencing were performed at the Institute for Research in Immunology and Cancer Genomics core facility. RNA-seq reads were aligned to the mouse reference genome (version GRCm38/mm10, gene annotation from Gencode version M13 based on Ensembl 88) with STAR aligner (Dobin et al., 2013). Read counts were normalized to reads per kilobase per million mapped reads (RPKM) and differential gene expression was calculated with DESeq2 (Love et al., 2014) using the RNA Express software suite (Illumina). Genes with a log_2_(fold change)>1 and *P*<0.05 were considered differentially expressed.

### Pathway analysis and hierarchical clustering

Pathway enrichment analysis of ranked gene lists was performed using the GSEA method (Subramanian et al., 2005). The mouse Molecular Signature database (Liberzon et al., 2011) collection of gene sets was used for GSEA search. Gene sets with a normalized enrichment score >1.4 and false discovery rate <0.1 are shown.

Transcriptome datasets of human HCC cell lines were downloaded from the Cancer Cell Line Encyclopedia (CCLE; https://sites.broadinstitute.org/ccle). mRNA abundance expressed in fragments per kilobase of transcript per million mapped reads (FPKM) was compared for human HCC cell lines, primary mouse hepatocytes and HepYF-M13 cells. Cluster heatmaps were generated using Morpheus (https://software.broadinstitute.org/morpheus) according to the transcriptional classification established by Caruso et al. (2019).

### Tumorigenesis assays

#### Subcutaneous injection

C57BL/6J mice were injected subcutaneously in the flank with 2×10^6^ HepYF cells resuspended in 100 μl of phosphate-buffered saline (PBS). Tumor volume was measured bi-weekly with a caliper using the formula V=(L×W×W)/2, where W is tumor width and L is tumor length.

#### Intrasplenic injection

HepYF-M13 cells (1×10^6^) resuspended in 50 μl of PBS were injected slowly into the spleen of C57BL/6J mice as described previously (Lacoste et al., 2017).

#### Intrahepatic injection

For sub-capsular hepatic injection, mice were anesthetized using 3% isoflurane for induction and 2% isoflurane for maintenance. After anesthesia, the abdominal area was shaved and surgical site antisepsis performed with three consecutives passages of iodine/alcohol. Abdominal incision was done from midline to left side, parallel with the last left rib (2 mm below the last rib). The left lateral liver lobe was exposed by gently rotating a sterile cotton-tipped applicator underneath. HepYF-M13 or Hepa 1-6 cells (1×10^6^) resuspended in 10 µl of PBS:Matrigel (1:1) were slowly injected underneath the hepatic capsule. The needle was retired after 30 s, followed by a gentle compression on the injection site with a sterile cotton-tipped applicator to minimize bleeding and the reflux of inoculated cells from the injection site. Surgical glue was also tried. The peritoneum was sutured with coated Vicryl thread and the skin with non-resorbable monofilament, followed by topical application of flamazine/lidocaine. Buprenorphine was administered at 1 mg/kg to ensure analgesia and 1 ml of saline solution was injected subcutaneously to avoid dehydration.

### Histology and IHC

Liver and subcutaneous tumors were fixed in 10% formalin, embedded in paraffin and sliced in 5-µm thin sections using a RM2255 microtome (Leica Biosystems). Tissue sections were mounted on glass slides and stained with Hematoxylin and Eosin using conventional protocols. IHC analysis was performed on the automated Ventana Discovery XT staining platform (Ventana Medical Systems). Slides were deparaffinized in xylene and hydrated in serial alcohol solutions. Antigen retrieval was performed by the heat-induced epitope retrieval method using standard citrate-based (pH 6) (H1) or EDTA-based (pH 9) (H2) epitope-retrieval methods on the BOND automated system (Leica Biosystems). The slides were incubated at room temperature for the indicated times with 100 µl of primary antibody. Bound primary antibodies were detected using either OmniMap anti-Rb HRP ChromoMap (Roche Diagnostics) with DAB detection kit (Ventana Medical Systems) or Biotin-SP-AffiniPure donkey anti-rabbit IgG (Jackson ImmunoResearch) with DABmap detection kit (Ventana Medical Systems). The slides were counterstained with Hematoxylin and coverslipped. After staining, the slides were scanned using the Hamamatsu NanoZoomer 2.0-HT digital pathology system. Sample preparation and histopathological analysis of mouse tissues were performed at the Institute for Research in Immunology and Cancer Histology core facility.

### *In vivo* pharmacology studies

C57BL/6J mice were injected subcutaneously with 2×10^6^ HepYF-M13 cells resuspended in 100 µl of PBS. Once the tumors reached a volume of 200-250 mm^3^, the mice were randomly divided into four groups: (1) vehicle (44% polyethylene glycol in H_2_O, pH 4.0) for the dasatinib group (*n*=8); (2) 30 mg/kg dasatinib group (*n*=8); (3) vehicle [12.5% Cremophore EL (Sigma-Aldrich) and 12.5% ethanol in H_2_O] for the sorafenib group (*n*=8); and (4) 30 mg/kg sorafenib group (*n*=8). Dasatinib and sorafenib were administered daily by oral gavage for 14 days. Tumor volumes were measured bi-weekly with a caliper. For the immunotherapy study, the mice were randomly assigned to two groups: (1) isotype IgG2a control at 200 µg/mouse (*n*=5); (2) anti-PD-1 at 200 µg/mouse (*n*=5). The antibodies were administered bi-weekly by intra-peritoneal injection for 14 days and the mice were sacrificed 10 days later.

### Statistical analysis

Data were analyzed using GraphPad Prism 5.0. A one-way ANOVA with Tukey's post hoc test was used to test differences in gene expression data between different groups. *P*-values >0.05 were considered statistically significant and are represented as follows: *P<0.05; **P<0.01; ***P<0.001; ****P<0.0001; and ns, not significant.

## Supplementary Material

10.1242/dmm.050553_sup1 Supplementary information

Table S2. GSEA of canonical pathways enriched in primary hepatocytes and HepYF-M13 cells

Table S3. GSEA of GO terms enriched in primary hepatocytes and HepYF-M13 cells
