# Supplementary material for: Syngeneic mouse model of YES-driven metastatic and proliferative hepatocellular carcinoma
Source: Dis Model Mech. 2024 Jul 25;17(7):dmm050553. doi: 10.1242/dmm.050553 (PMC11552496; doi:10.1242/dmm.050553)
Supplement: Supplementary information [file dmm-17-050553-s1.pdf]

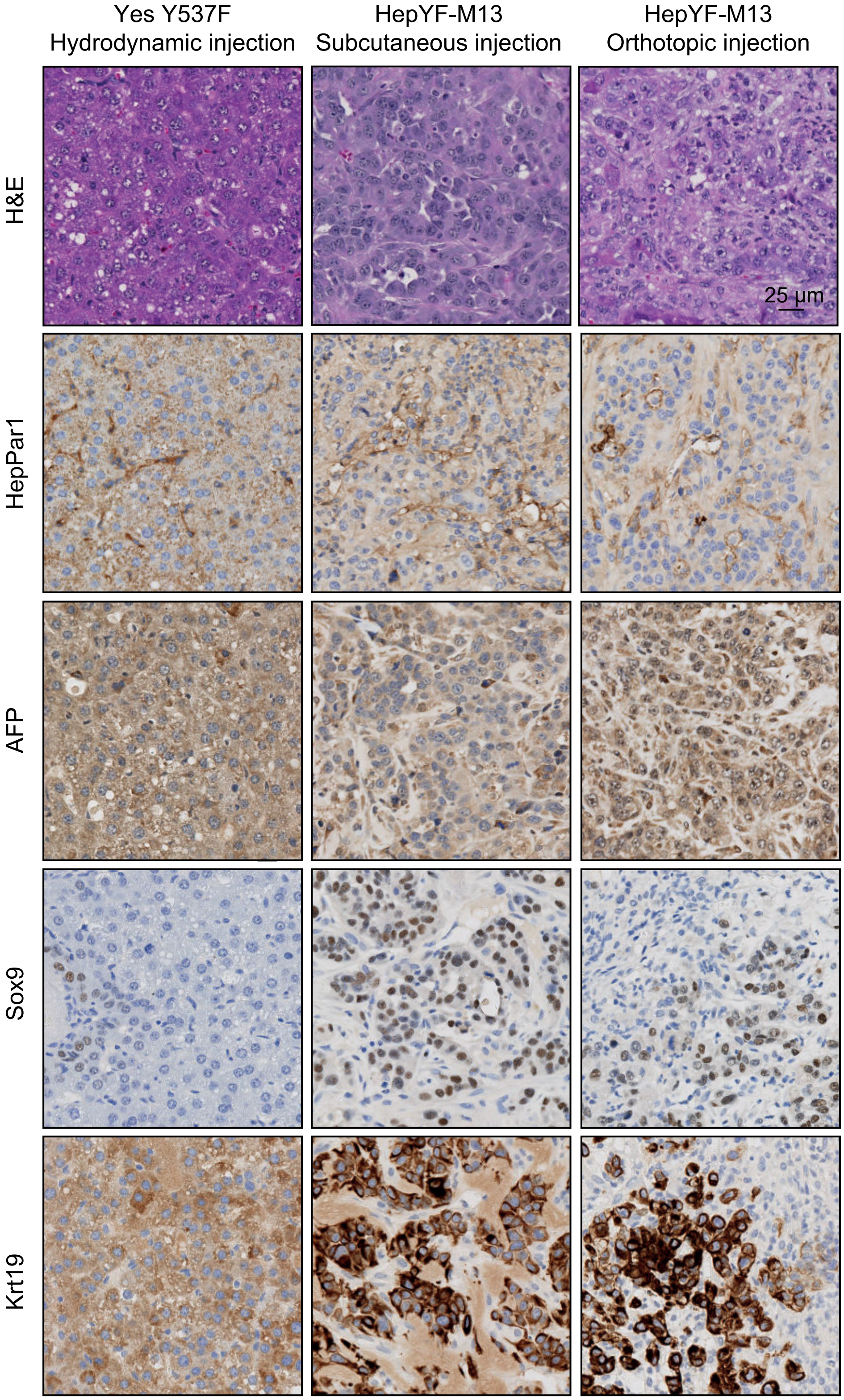

Fig. S1. Comparative histopathological analysis of liver tumors generated by hydrodynamic injection of YES Y537F and by subcutaneous or intrahepatic injection of HepYF-M13 cells. Liver tumors were generated as described in Materials and Methods. Representative H/E staining and immunohistochemistry staining for HepPar1, AFP, Sox9 and Krt19 in liver tumor sections.

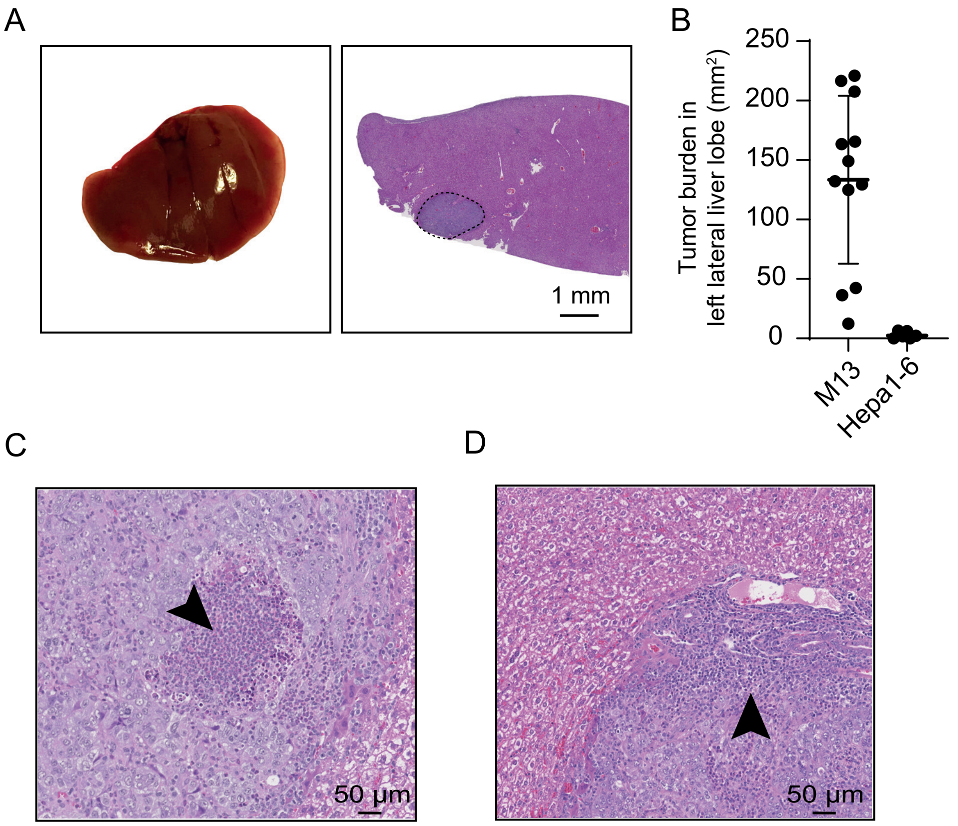

Fig. S2. Histological analysis of the mouse Hepa 1-6 orthotopic HCC model. Hepa 1-6 cells were injected in the liver parenchyma of C57BL/6J mice (n = 6). (A) Left, representative image of the liver at sacrifice 8 days post-inoculation. Right, representative low-power photomicrograph of a liver section stained with H/E showing a small tumor nodule at the site of injection (dashed line). (B) Area of tumor nodules measured on H&E-stained liver sections. For comparison, areas of tumor lesions measured in the HepYF-M13 model are shown and are a replication of the data reported in Fig. 6C. Data are means SD. (C) Photomicrograph of a liver tumor section showing the presence of a necrotic microabscess (arrowhead). (D) Photomicrograph showing an inflammatory reaction at the tumor-hepatocyte interface (arrowhead).

Disease Models & Mechanisms • Supplementary information

Table S1. Efficiency of liver tumor cells allografts

	Subcutaneous injection	
**Cell population**	**NSG**	**C57BL/6J**
B6-1	1/3	0/3
B6-2	0/3	0/3
B6-3	1/3	0/3
B6-4	3/3	0/3
B6-5	3/3	0/3
B6-6	1/3	0/3

	Subcutaneous injection	
**Cell population**	**NSG**	**C57BL/6J**
B6-3 TT M20	3/3	3/3
B6-4 TT M33	3/3	3/3
B6-4 TT M35	3/3	2/3
B6-5 TT M13	3/3	2/3
B6-5 TT M14	3/3	3/3

Table S2. GSEA of canonical pathways enriched in primary hepatocytes and HepYF-M13 cells

Available for download at
https://journals.biologists.com/dmm/article-lookup/doi/10.1242/dmm.050553#supplementary-data

Table S3. GSEA of GO terms enriched in primary hepatocytes and HepYF-M13 cells

Available for download at
https://journals.biologists.com/dmm/article-lookup/doi/10.1242/dmm.050553#supplementary-data

Table S4. Primer sequences used for real-time quantitative PCR analysis

Gene	Ref	Forward primer	Reverse primer	Probe (Roche)
Actb	NM_007393.3	aaggccaaccgtgaaaagat	gtggtacgaccagaggcatac	56
Afp	NM_007423.4	tggatgtcaggacaatctgg	gcagctttgcttggacagt	91
Alb	NM_009654.4	caacaaggagtgctgccatg	aagttccgccctgtcatctg	3
Arg1	NM_007482.3	gaatctgcatgggcaacc	gaatcctggtacatctgggaac	2
Cd24a	NM_009846.2	ctggggttgctgcttctg	caacagatgtttggttgcagtaa	68
Cd44	NM_009851.2, NM_001039150.1, NM_001039151.1, NM_001177785.1, NM_001177786.1, NM_001177787.1	ttccagaggcgactagatcc	gcggcaggttacattcaaa	42
Cps1	NM_001080809.2	cagttccacccagaggtcag	gtaatggtggtgcctttgcc	7
Gapdh	NM_008084.2	tgtccgtcgtggatctgac	cctgcttcaccaccttcttg	80
Ggt1	NM_001305992.1, NM_001379537.1, NM_001379538.1, NM_001379539.1, NM_001379540.1, NM_001379541.1, NM_008116.3	acctgtctgcggtttcagag	caggaccttggagccaaagt	5
Glul	NM_008131.5	cgacttttctgccggtgttg	ggccgacggtcttcaaagta	2
Golm1	NM_027307.4, NM_001035122.2	ccaggatgagaaggcggttt	tcctctgcagtgccttcaac	9
Gpc3	NM_016697.3	atgtgcagaagaacggaggc	atattggcgttgctgggagt	77
Hnf4a	NM_001312906.1 NM_001312907.1 NM_001312907.1	ccaagaggtccatggtgttt	ccgagggacgatgtagtcat	68
Hprt	NM_013556.2	tcctcctcagaccgctttt	cctggttcatcatcgctaatc	95
Krt19	NM_008471.2	tgacctggagatgcagattg	cctcagggcagtaatttcctc	17
Krt7	NM_033073	ggagatggccaaccacag	ggcctggagtgtctcaaactt	41
Prom1	NM_008935	gccagcaagatctgcgata	tctatccactgatgggagctg	32
Sox4	NM_009238.3	ggctgcatcgttctctcc	gagtccgcaccttgtagacg	74
Sox9	NM_011448.4	cagcaagactctgggcaag	atcggggtggtctttcttgt	25
Spp1	NM_009263	cccggtgaaagtgactgatt	ttcttcagaggacacagcattc	82
Tspan8	NM_001168679.1, NM_001168680.1, NM_146010.2	gaaagaaagccggtgcatgc	aggtttgaaagcggctccta	74

Disease Models & Mechanisms • Supplementary information
